# Supplementary material for: Bacmid Expression of Granulovirus Enhancin En3 Accumulates in Cell Soluble Fraction to Potentiate Nucleopolyhedrovirus Infection
Source: Viruses. 2021 Jun 25;13(7):1233. doi: 10.3390/v13071233 (PMC8309998; doi:10.3390/v13071233)
Supplement: Supplementary file 1 [file viruses-13-01233-s001.zip › viruses-1262242-supplementary.pdf]

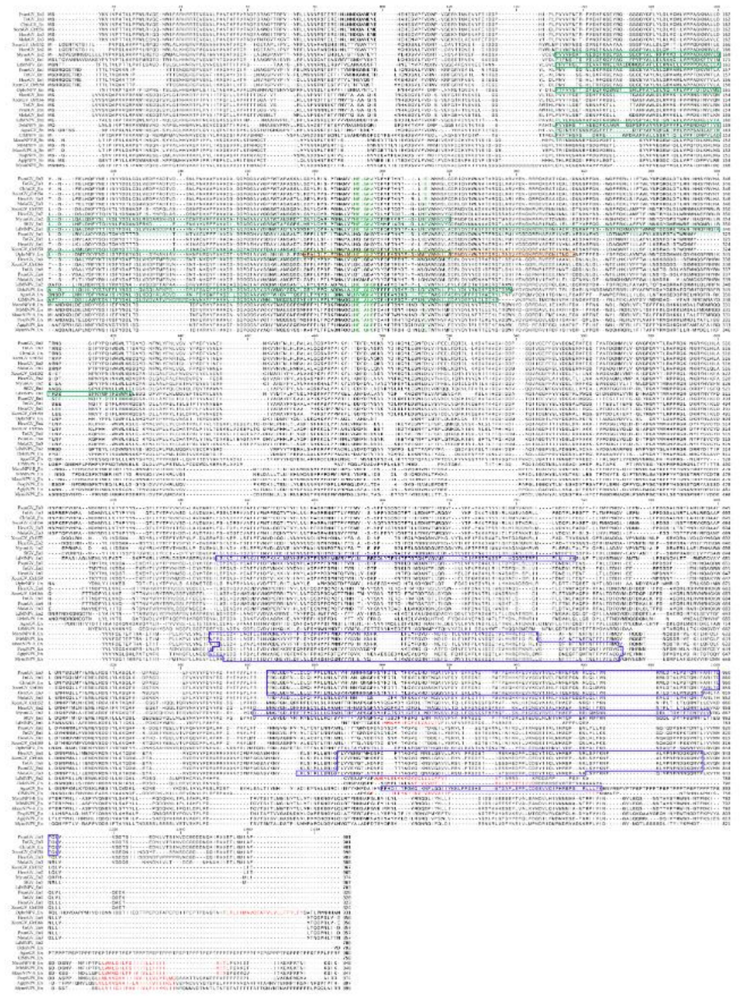

Figure S1. Global alignment of enhancin proteins. The HEXXHX(8,28)E motif is represented with light green residues. The transmembrane helices are represented in red residues. The Peptidase_M60 Superfamily, the SslE_AcfD_Zn_LP and Mucin_bdg super family domains are shown in green, orange, and blue boxes, respectively.
